# Racial and Ethnic Inequalities in Actual vs Nearest Delivery Hospitals

**DOI:** 10.1001/jamanetworkopen.2025.1404

**Published:** 2025-03-21

**Authors:** Nansi S. Boghossian, Lucy T. Greenberg, Jeffrey S. Buzas, Joshua Radack, Molly Passarella, Jeannette Rogowski, George R. Saade, Ciaran S. Phibbs, Scott A. Lorch

**Affiliations:** 1 Department of Epidemiology and Biostatistics, Arnold School of Public Health, University of South Carolina, Columbia; 2 Vermont Oxford Network, Burlington; 3 Department of Mathematics and Statistics, University of Vermont, Burlington; 4 Department of Pediatrics, University of Pennsylvania School of Medicine, Philadelphia; 5 Department of Health Policy and Administration, The Pennsylvania State University, State College; 6 Department of Obstetrics & Gynecology, Eastern Virginia Medical School, Norfolk; 7 Health Economics Resource Center and Center for Implementation to Innovation, Veterans Affairs Palo Alto Health Care System, Menlo Park, California; 8 Departments of Pediatrics and Health Policy, Stanford University School of Medicine, Stanford, California; 9 Leonard Davis Institute of Health Economics, Wharton School, University of Pennsylvania, Philadelphia

## Abstract

**Importance:**

Minoritized racial and ethnic groups, such as American Indian and Black individuals, often receive lower quality health care compared with White individuals. There is limited understanding of how these disparities extend to obstetric care, particularly when comparing the quality of care at the actual delivery hospital vs the nearest obstetric hospital based on the birthing individual’s residence.

**Objective:**

To examine inequality in care based on the actual delivery hospital and the closest delivery hospital to the birthing individual’s residential zip code centroid.

**Design, Setting, and Participants:**

This population-based retrospective cohort study used data from 5 states (2008 to 2020 for Michigan, Oregon, and South Carolina; 2008 to 2018 for Pennsylvania; and 2008 to 2012 for California). Individuals delivering a fetal death or a live birth with gestational age between 22 to 44 weeks were included. Analysis was conducted between February and August 2024.

**Exposure:**

Race and ethnicity.

**Main Outcomes and Measures:**

The obstetric inequality index was calculated using Gini coefficients from Lorenz curves for American Indian, Asian, Black, and Hispanic birthing individuals compared with White individuals, with hospitals ranked by their standardized morbidity ratio for nontransfusion severe maternal morbidity.

**Results:**

There were 6 418 635 birthing individuals across 549 hospitals (23 050 American Indian individuals [0.4%], 463 342 Asian individuals [7.2%], 807 738 Black individuals [12.6%], 1 645 922 Hispanic individuals [25.6%], and 3 279 315 White individuals [51.1%]). Compared with White individuals, American Indian and Black individuals delivered at lower-quality hospitals, while there was no significant difference for Asian and Hispanic individuals (delivery hospital inequality index: American Indian, 0.07 [95% CI, 0.03 to 0.11]; Asian, −0.02 [95% CI, −0.08 to 0.04]; Black, 0.15 [95% CI, 0.12 to 0.19]; Hispanic −0.04 [95% CI, −0.09 to 0.01]). Black individuals lived closer to lower-quality hospitals than White individuals (closest hospital inequality index for Black individuals: 0.11 [95% CI, 0.07 to 0.14]). Asian and Hispanic individuals had similar closest hospital inequality indices to White individuals. The inequality index for Black individuals would have been lower if individuals had delivered at their nearest hospital.

**Conclusions and Relevance:**

This cohort study found that American Indian and Black individuals delivered at lower-quality hospitals than White individuals. The disparity in care between Black and White birthing individuals would have been reduced if individuals had delivered at their nearest hospital.

## Key Points

**Question:**

What is the extent of care inequality based on the actual delivery hospital and the closest delivery hospital to the birthing individual’s residential zip code centroid?

**Findings:**

In this cohort study of 6 418 635 birthing individuals from 5 states, American Indian and Black individuals delivered at lower-quality hospitals compared with White individuals, while there was no significant difference for Asian and Hispanic individuals. The disparity between Black and White individuals would have been reduced if all patients had delivered at their nearest hospital.

**Meaning:**

These findings indicate significant racial disparities in hospital quality, likely reflecting broader issues in health care access and availability.

## Teaser

This cohort study examines inequality in perinatal care based on the actual delivery hospital and the closest delivery hospital to the birthing individual’s residential zip code centroid.

## Introduction

The US is experiencing a maternal health crisis, with maternal mortality and severe maternal morbidity (SMM) affecting around 700 and 60 000 birthing individuals annually, respectively.^1^ Alarmingly, these numbers are increasing.^2,3^ American Indian and Black individuals bear a disproportionate burden of maternal mortality and SMM.^3,4,5,6,7,8,9,10,11^ The drivers for these disparities include higher rates of pregnancy complications, medical conditions,^12^ racism,^13^ stress,^14^ and potentially lower quality of health care.^7^ Several studies have also linked quality of care with racial and ethnic disparities in obstetrics.^7,15,16,17,18^ However, despite extensive research, there is limited understanding of how these racial and ethnic disparities extend to obstetric care, particularly regarding the quality of care at the delivery hospital vs the nearest obstetric hospital based on the birthing individual’s residence.

This study examines the inequality in care for American Indian, Asian, Black, and Hispanic birthing individuals compared with White birthing individuals in 5 US states. We defined inequality as the concentration of racial or ethnic groups in lower-quality hospitals, using Gini coefficients and Lorenz curves. To distinguish between the services reasonably available to patients and their actual delivery choices, we examined disparities in care based on 2 factors: (1) the hospital where delivery actually occurred and (2) the closest hospital to the birthing individual’s residential zip code. This approach is grounded in prior data suggesting that pregnant patients often deliver at the nearest hospital with delivery services.^19,20,21^ Thus, the quality of care at this closest hospital may serve as a proxy for the care available within that zip code.

## Methods

This cohort study was approved by the institutional review boards of the Children’s Hospital of Philadelphia, University of South Carolina, and the health departments in each of the included states with a waiver of informed consent because the study was determined non–human participant research, since the records were deidentified. We followed the Strengthening the Reporting of Observational Studies in Epidemiology (STROBE) reporting guideline.

### Study Sample

We used birth and fetal death certificates with gestational ages between 22 and 44 weeks, linked to maternal hospital records^22^ from California, Michigan, Oregon,^23^ Pennsylvania, and South Carolina. The study period included 2008 to 2020 for Michigan, Oregon, and South Carolina; 2008 to 2018 for Pennsylvania; and 2008 to 2012 for California.

### Study Variables

Maternal race and ethnicity on the birth or fetal death certificate were based on self-identification and were categorized into American Indian, Asian, non-Hispanic Black (hereafter, *Black*), Hispanic, non-Hispanic White (hereafter, *White*), and other or multiracial. Race and ethnicity were assessed given their associations with health care quality.^7,15,16,17,18^

Nontransfusion SMM was defined using the Centers for Disease Control and Prevention *International Classification of Diseases, Ninth Revision *(*ICD-9*) or *International Statistical Classification of Diseases and Related Health Problems, Tenth Revision *(*ICD-10*) diagnosis and procedure code algorithm, which includes 16 life-threatening maternal conditions and 4 lifesaving procedures.^24^ We used nontransfusion SMM because the number of transfused units is unavailable and blood transfusion alone is not indicative of SMM severity. To facilitate bridging the transition from *ICD-9* to *ICD-10*, we used the Maternal and Child Health Bureau federally available data resource document.^25^

### Adjustment Variables

Adjustment variables included maternal characteristics (age, parity, prenatal care adequacy, delivery mode), sickle cell disease, gestational age, and hospital level of obstetric care and volume. Other comorbidities were adjusted for using 26 of 27 variables from the obstetric comorbidity index (eTable 1 in Supplement 1), excluding placenta accreta spectrum, as it almost always results in SMM. Maternal age was included as a quadratic variable, and gestational age was modeled linearly as a continuous variable.

### Actual vs Nearest Delivery Hospital

For each birthing individual, we calculated driving time to the actual delivery hospital and to the nearest delivery hospital from the centroid of maternal residential zip code. The actual delivery hospital was identified based on maternal hospital discharge data. Maternal residential zip code was based on birth certificate data. We used Google Maps (Alphabet) to calculate optimal driving times from the centroid of the maternal residential zip code to hospital addresses obtained from American Hospital Association data. A hospital was identified as a delivery hospital if it had at least 10 deliveries per year.

### Statistical Analysis

We conducted descriptive analyses examining the sociodemographic and clinical characteristics of Asian, American Indian, Black, Hispanic, and White birthing individuals. Statistical significance was set at α = .05. All analyses were conducted using R software version 4.0.5 (R Project for Statistical Computing) from February to August 2024.

#### Hospital Quality

eFigure 1 in Supplement 1 shows the distribution of hospital nontransfusion SMM rates by state. Hospital quality for a given hospital-year was defined by the hospital-level standardized morbidity ratio (SMR) for nontransfusion SMM for the previous 3-year period. We estimated SMRs using hierarchical logistic regression models fit for each state and rolling 3-year period and adjusted for the aforementioned covariates. Unadjusted and adjusted SMRs for a 3-year period (2008-2010) are provided in eFigure 2 in Supplement 1. SMRs were estimated as the ratio of predicted to expected SMM events, where the numerator is the model-predicted number of SMM events for a hospital given its volume and case mix, and the denominator is the expected number of SMM cases of an average-quality hospital with the same case mix. These SMRs measure risk-adjusted hospital quality relative to other hospitals in the same state and time period.

#### Lorenz Curves and Hospital Inequality Indices

To develop Lorenz curves for inequality, we ranked delivery hospitals by SMRs and we plotted the cumulative percentage of White individuals (x-axis) against the cumulative percentage of individuals with minoritized race and ethnicity (American Indian, Asian, Black, or Hispanic; y-axis). If all delivery hospitals had identical SMRs or if the proportion of each racial and ethnic group was distributed equally across low- and high-quality hospitals, this would coincide with the diagonal line of equality. A curve below the diagonal line reflects a high concentration of individuals with minoritized race and ethnicity attending disproportionately poorer-quality hospitals compared with White individuals. Conversely, a curve above the diagonal line indicates that these individuals attended higher-quality hospitals.

The Gini coefficient is proportional to the area between the diagonal and the curve. When the curve is above the diagonal, this area is included as a negative value. Larger Gini values reflect greater disparities in hospital quality between White individuals and those with minoritized race and ethnicity. A Gini coefficient of 0 indicates no difference in hospital quality, while a coefficient of 1 means all White individuals received care at higher-quality hospitals compared with those with minoritized race and ethnicity. Conversely, a Gini coefficient of −1 suggests that all individuals with minoritized race and ethnicity received care at higher-quality hospitals compared with White individuals. This method follows the approach used by Farell et al^26^ in measuring income inequality within residential neighborhoods.

We generated Lorenz curves to assess inequality among American Indian, Asian, Black, and Hispanic birthing individuals, using White individuals as the reference group. These curves combined data across all states and years and were also analyzed separately by insurance type (government vs commercial) and by state. We defined the delivery hospital inequality index for each racial and ethnic group as the Gini coefficient associated with each curve. We obtained CIs by bootstrap resampling by hospital. Next, we ranked patients by the SMR of the nearest delivery hospital to their residential zip code centroid and created Lorenz curves and their associated Gini coefficients, defined as the closest hospital inequality indices. These curves and indices illustrate disparities in access to quality hospitals among racial and ethnic groups, based on driving time from their residential zip code centroid.

As a secondary analysis, we calculated the difference in quality percentiles between the actual delivery hospital and the nearest hospital to the residential zip code centroid for individuals who did not deliver at their nearest delivery hospital. We then compared these differences across racial and ethnic groups.

## Results

The final sample included 6 418 635 birthing individuals across 549 hospitals. Exclusions were made for 4098 individuals with placenta accreta spectrum, 1526 deliveries in hospitals without obstetric units, 21 317 deliveries in hospitals with fewer than 3 consecutive years of obstetric service, and 199 268 individuals identified as other race or ethnicity or multiracial (eFigure 3 in Supplement 1). The racial and ethnic distribution was 23 050 American Indian individuals (0.4%), 463 342 Asian individuals (7.2%), 807 738 Black individuals (12.6%), 1 645 922 Hispanic individuals (25.6%), and 3 279 315 White individuals (51.1%). The Table shows maternal characteristics by race and ethnicity. American Indian, Black, and Hispanic individuals were generally younger and more likely to have government insurance, whereas Asian and White individuals tended to be older and more likely to have commercial insurance. American Indian individuals had the highest prevalence of diabetes, Asian individuals had the highest rates of gestational diabetes, and Black individuals had the highest rates of preeclampsia and gestational hypertension. Black individuals had the highest median SMM comorbidity index score. Inadequate prenatal care utilization was most prevalent among American Indian and Black individuals.

**Table.** Maternal Characteristics by Race and Ethnicity Across All 5 States

Characteristic	Individuals, No. (%)					
	American Indian (n = 23 050)	Asian (n = 463 342)	Black (n = 807 738)	Hispanic (n = 1 645 922)	White (n = 3 279 315)
Maternal age, y					
<20	2622 (11.4)	7251 (1.6)	101 998 (12.6)	201 199 (12.2)	159 838 (4.9)
20-24	6714 (29.1)	38 946 (8.4)	257 638 (31.9)	440 801 (26.8)	616 435 (18.8)
25-34	11 111 (48.2)	291 502 (62.9)	361 120 (44.7)	783 382 (47.6)	1 950 515 (59.5)
≥35	2603 (11.3)	125 643 (27.1)	86 982 (10.8)	220 540 (13.4)	552 527 (16.8)
Gestational age at delivery, median (IQR), wk	39 (38-40)	39 (38-40)	39 (38-40)	39 (38-40)	39 (38-40)
Smoker^a^	4137 (29.4)	2896 (2.1)	79 866 (11.9)	29 123 (8.1)	463 075 (17.8)
Maternal insurance					
Commercial	7987 (34.7)	329 363 (71.1)	245 922 (30.4)	493 025 (30.0)	2 146 330 (65.5)
Government	14398 (62.5)	114 306 (24.7)	552 567 (68.4)	1 120 466 (68.1)	1 081 941 (33.0)
Self-pay	430 (1.9)	17 086 (3.7)	7217 (0.9)	25 793 (1.6)	36 526 (1.1)
Other	233 (1.0)	2547 (0.5)	1960 (0.2)	6557 (0.4)	14 346 (0.4)
Maternal education					
No high school	627 (2.7)	7741 (1.7)	7268 (0.9)	217 845 (13.2)	30 374 (0.9)
Some high school	4680 (20.3)	17 643 (3.8)	138 511 (17.1)	406 392 (24.7)	238 825 (7.3)
High school diploma or GED	7673 (33.3)	65 484 (14.1)	282 565 (35.0)	497 617 (30.2)	727 546 (22.2)
At least some college	7134 (31.0)	91 264 (19.7)	273 780 (33.9)	346 218 (21.0)	1 001 829 (30.5)
4-y college	1608 (7.0)	155 410 (33.5)	61 013 (7.6)	98 167 (6.0)	788 738 (24.1)
>4-y college	905 (3.9)	110 544 (23.9)	30 381 (3.8)	39 425 (2.4)	454 856 (13.9)
Missing	423 (1.8)	15 256 (3.3)	14 220 (1.8)	40 258 (2.4)	37 147 (1.1)
Cesarean delivery	7629 (33.1)	150 449 (32.5)	270 545 (33.5)	517 278 (31.4)	1 040 607 (31.7)
Preeclampsia	617 (2.7)	6758 (1.5)	23 095 (2.9)	35 243 (2.1)	77 022 (2.3)
Gestational hypertension	932 (4.0)	9674 (2.1)	44 875 (5.6)	39 555 (2.4)	154 521 (4.7)
Gestational diabetes	1792 (7.8)	60 242 (13.0)	43 113 (5.3)	132 519 (8.1)	208 161 (6.3)
Diabetes	428 (1.9)	4333 (0.9)	13 425 (1.7)	19 448 (1.2)	28 667 (0.9)
SMM comorbidity index score with transfusion, median (IQR)	2 (0-8)	0 (0-3)	3 (0-9)	0 (0-4)	1 (0-5)
SMM comorbidity index nontransfusion score, median (IQR)	0 (0-7)	1 (0-4)	4 (0-12)	0 (0-6)	0 (0-7)
Multiple gestation	331 (1.4)	7609 (1.6)	16 859 (2.1)	19 655 (1.2)	65 994 (2.0)
Adequacy of Prenatal Care Utilization Index					
Inadequate	4159 (18.0)	37 996 (8.2)	154 743 (19.2)	197 971 (12.0)	282 646 (8.6)
Intermediate	2408 (10.4)	52 050 (11.2)	105 945 (13.1)	171 441 (10.4)	421 576 (12.9)
Adequate	8196 (35.6)	203 494 (43.9)	250 555 (31.0)	681 627 (41.4)	1 416 094 (43.2)
Adequate plus	6902 (29.9)	151 512 (32.7)	244 939 (30.3)	524 877 (31.9)	1 034 510 (31.5)
Missing	1385 (6.0)	18 290 (3.9)	51 556 (6.4)	70 006 (4.3)	124 489 (3.8)
Prepregnancy BMI >30	7514 (34.3)	33 651 (7.8)	269 979 (35.9)	406 290 (26.4)	718 000 (22.9)

Abbreviations: BMI, body mass index (calculated as weight in kilograms divided by height in meters squared); SMM, severe maternal morbidity.

^a^ Smoking status missing in California.

**Figure 1.**
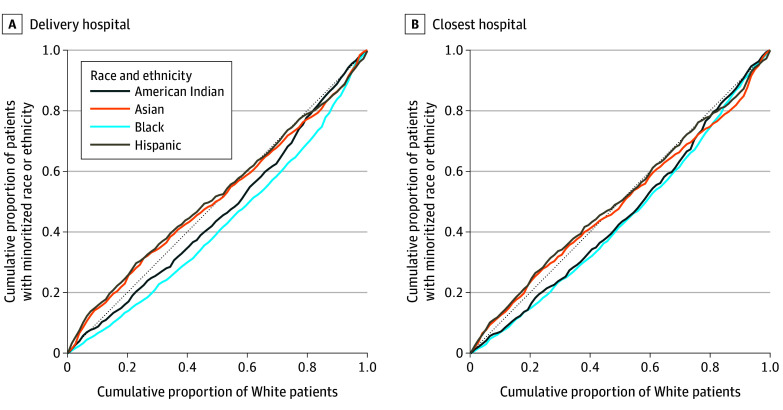
Lorenz Curves for Inequality by Actual Delivery Hospital and Closest Obstetric Hospital Across 5 States

Figure 1 shows the Lorenz curves for inequality by actual delivery hospitals and nearest hospitals across all 5 states. The actual delivery hospital inequality indices were −0.02 (95% CI, −0.08 to 0.04) for Asian individuals and −0.04 (95% CI, −0.09 to 0.01) for Hispanic individuals, indicating no statistically significant difference in quality of hospitals compared with White individuals. The indices were 0.07 (95% CI, 0.03 to 0.11) for American Indian individuals and 0.15 (95% CI, 0.12 to 0.19) for Black individuals, indicating lower-quality hospitals compared with White individuals.

The closest-hospital inequality indices were 0.01 (95% CI, −0.05 to 0.06) for Asian individuals and −0.02 (95% CI, −0.08 to 0.04) for Hispanic individuals, indicating no significant differences in closest-hospital quality. The closest-hospital inequality indices were 0.08 (95% CI, 0.04 to 0.14) for American Indian individuals and 0.11 (95% CI, 0.07 to 0.14) for Black individuals, with curves below the diagonal, indicating that these individuals lived closer to lower-quality hospitals than White individuals. The inequality index for Black individuals would have been lower if they had delivered at the nearest hospital rather than at the actual delivery hospital. For American Indian, Asian, and Hispanic individuals, the closest delivery hospital had worse quality indices than the actual delivery hospital, although the CIs largely overlapped (Figure 1).

The proportions of American Indian, Asian, Black, Hispanic, and White birthing individuals by quintiles of ranked SMRs based on the actual delivery hospital and the closest delivery hospital are shown in eFigure 4 in Supplement 1. Black individuals were more likely to be concentrated at lower-quality obstetric hospitals, while Asian and Hispanic individuals were more likely to be concentrated at higher-quality hospitals.

Lorenz curves by insurance type (government vs commercial) are presented in eFigure 5 in Supplement 1. No significant differences in inequality indices by insurance type were found, except that Black individuals with private insurance lived closer to slightly lower quality hospitals compared with Black individuals with government insurance.

**Figure 2.**
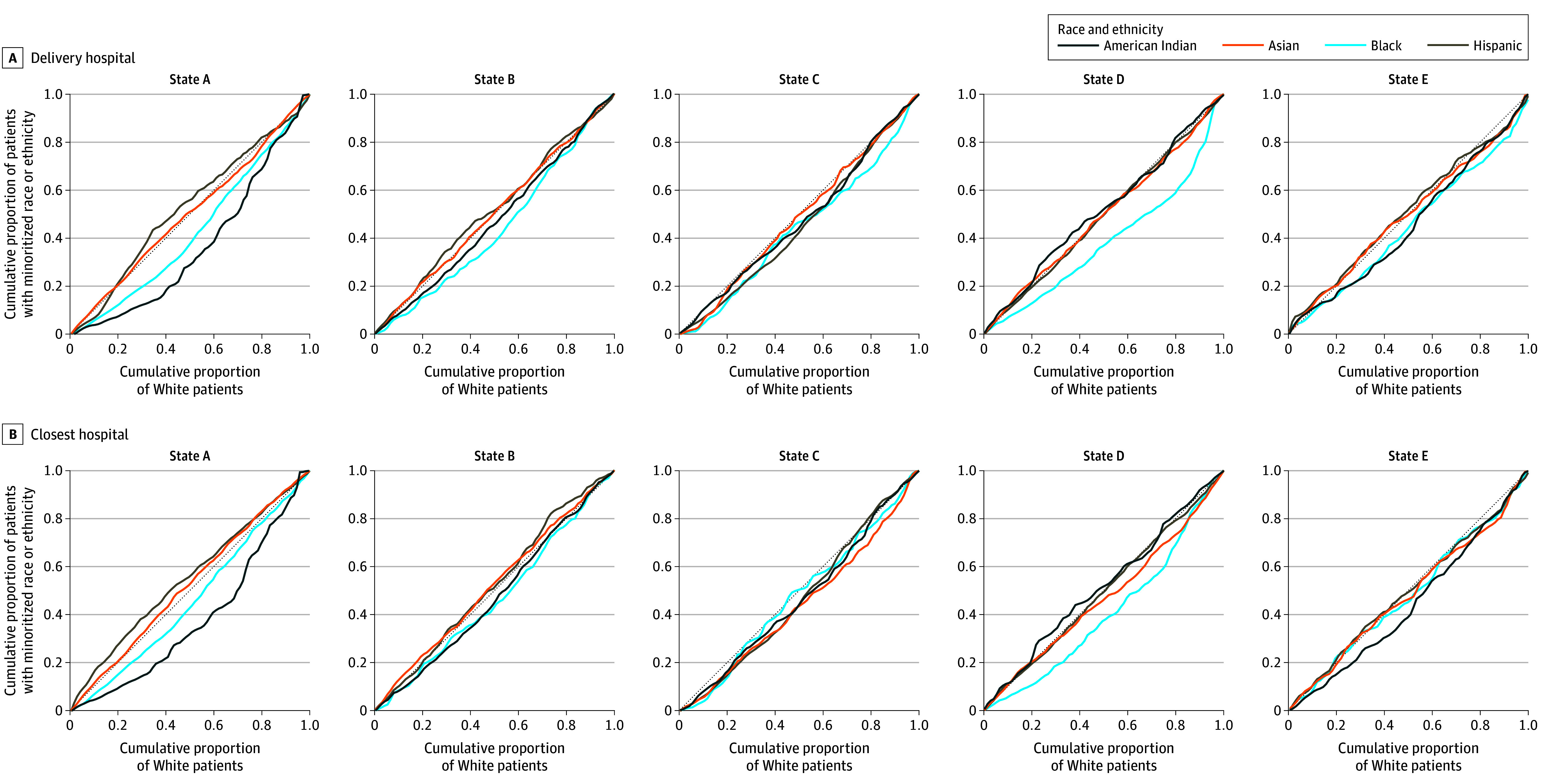
Lorenz Curves for Inequality by Actual Delivery Hospital and Closest Obstetric Hospital by State

Figure 2 shows Lorenz curves for inequality by actual delivery hospital and closest hospital for each state. In all states, Black individuals delivered at lower-quality hospitals than White individuals. In states A and E, American Indian individuals also delivered at lower-quality hospitals. In state C, Hispanic individuals delivered at lower-quality hospitals. If all birthing individuals had delivered at their nearest hospital, disparities in care between Black and White birthing individuals would have been reduced in all states, and in states B, C, and E, there would be no significant differences in hospital quality between Black and White individuals. If individuals in states C and D delivered at the closest hospital, the disparity between Asian and White individuals would have been worse than that based on the actual delivery hospital. There were no major differences in the inequality of care for American Indian and Hispanic individuals when comparing actual and closest delivery hospitals.

**Figure 3.**
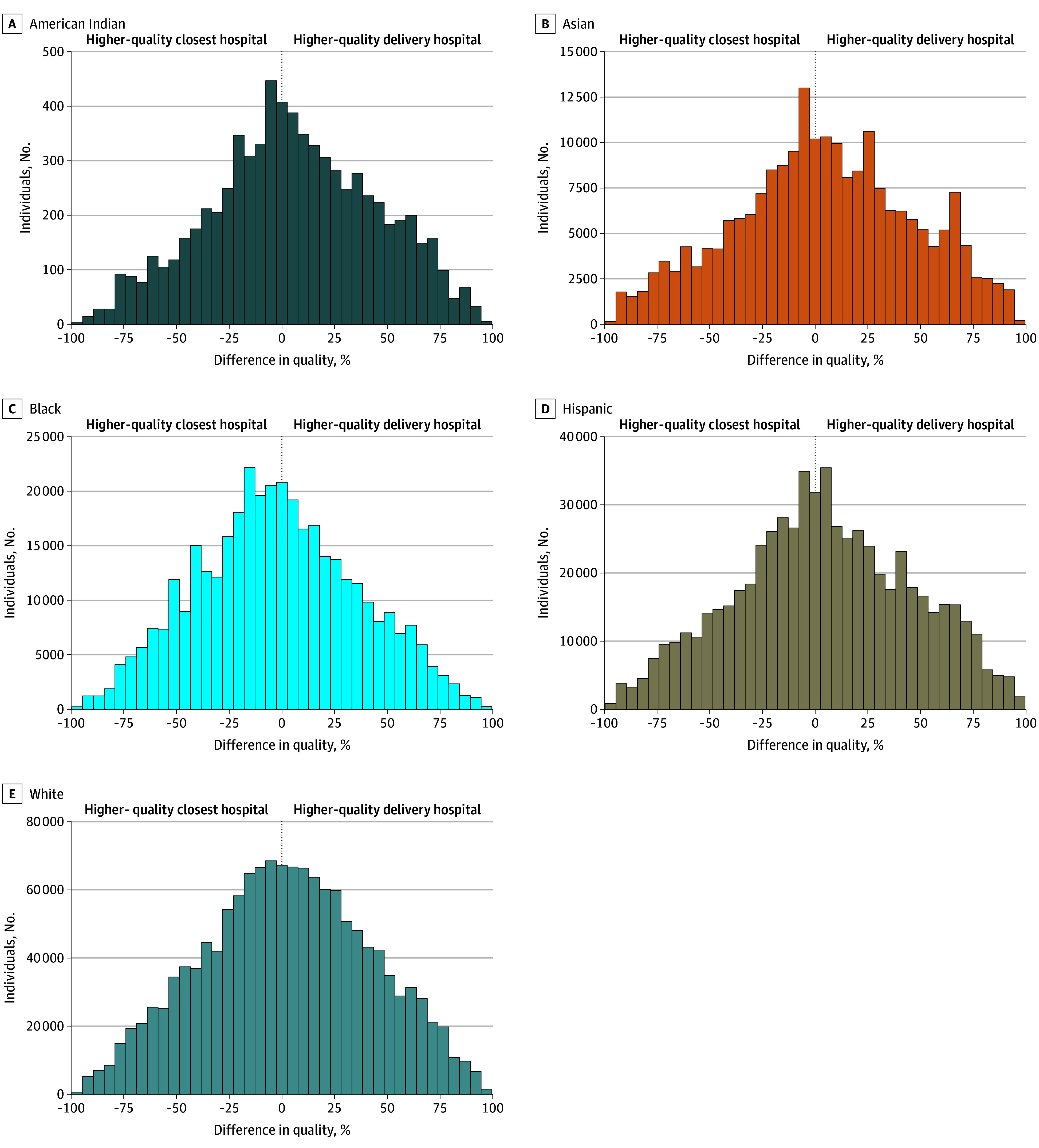
Quality Percentile Differences Between Actual and Nearest Hospitals, by Race and Ethnicity, for Deliveries at Nonnearest Hospitals

Figure 3 displays the distributions of differences in quality percentiles between the actual and nearest delivery hospitals, stratified by racial and ethnic groups, for individuals who did not deliver at their nearest hospital. More than half (202 216 individuals [54.0%]) of Black individuals who did not deliver at their nearest hospital delivered at a hospital with a lower quality ranking, and nearly one-third (120 222 individuals [32.1%]) delivered at a hospital ranked more than 20 percentage points lower than their nearest hospital. Among American Indian, Asian, Hispanic, and White individuals, 46% to 48% delivered at hospitals with lower quality rankings, and 26% to 29% delivered at hospitals ranked more than 20 percentage points lower than their nearest hospital.

## Discussion

In this population-based cohort study of 6.4 million birthing individuals in 5 states, we found that American Indian and Black individuals delivered at lower-quality hospitals compared with White individuals. The disparity in care between Black and White individuals would have been reduced if individuals delivered at the closest delivery hospital. State differences in care inequality were observed, with 3 of 5 states having no differences in care quality between Black and White individuals if deliveries occurred at the closest hospital.

In the perinatal field, few studies have linked quality of care with racial disparities. Data from New York, New York, suggest that the quality of the delivery hospital, measured using risk-standardized SMM rates, explained 47.7% of the disparity in SMM rates between Black and White patients^7^ and 37% of the disparity in SMM rates between Hispanic and White patients.^15^. In California, hospital choice explained only 7.8% of the SMM variation between Black and White individuals and 16.1% to 24.2% for other racial and ethnic groups.^18^ In few other studies, hospitals serving mostly Black patients in several states performed worse on several delivery-related indicators^16^ and risk-adjusted SMM.^17^ Inequality of care has also been documented among Black infants, with Black infants with very low birth weight receiving care at neonatal intensive care units with a lower Baby-MONITOR score, a composite of 5 process and 4 outcome measures, compared with White infants with very low birth weight.^27^

Interestingly, we observed disparities across states and racial and ethnic groups in health care inequality based on both the actual and the nearest delivery hospital to the birthing individual’s residential zip code. In all 5 states in our study, Black birthing individuals lived closer to higher-quality hospitals than the hospitals they chose for delivery. The reasons that pregnant individuals select certain hospitals are not well understood. Area-level factors, such as geographic proximity, structural racism, insurance status, and comorbidities, might be determinants of access to care. The percentage of office-based physicians accepting new Medicaid patients (69%) for example, has been shown to be lower than the percentage accepting new Medicare patients (84%) or new privately insured patients (85%),^28^ indicating that insurance type might limit access to certain hospitals that differ on quality of care provided. Safety-net hospitals treating predominantly poor or Medicaid patients have been shown to have lower adherence to the quality of cardiac, pneumonia, and colon cancer care measures than non–safety-net hospitals.^29,30,31,32^ Differences in geographic proximity might be another determinant of hospital quality access.^33,34^ Limited research among nonpregnant adults undergoing surgery and treatment for myocardial infarction also suggests that residential segregation amplifies the association between geographic proximity and hospital selection.^35,36^ Black Medicare patients were more likely than White Medicare patients to bypass nearby high-quality hospitals and use low-quality ones if they lived in highly segregated regions.^35,36^ In contrast, in regions with less segregation, Black and White individuals showed equal likelihood of seeking care at low-quality hospitals.^35,36^

Among birthing individuals, studies investigating the factors influencing hospital choice during delivery are limited. One study conducted in California in 1985 found that hospital proximity, hospital quality, maternal risk status, and insurance type were associated with hospital choice.^21^ Another study examined the associations of geographic proximity and maternal characteristics, including race, with the choice of delivery hospital among mothers of very low birth weight newborns in New Yorky, New York (1996-2001), and found that geography played some part, but proximity alone did not elucidate underuse of good quality hospitals with low neonatal mortality rates.^19^ Compared with White birthing individuals, Black birthing individuals were more likely to bypass a high-quality hospital when it was in their neighborhood and less likely to use a high-quality hospital if they delivered outside their neighborhood.^19^ Maternal characteristics were also instrumental in the place of delivery, with geographic proximity being more important to the birthing individuals who were less educated, had Medicaid or no insurance, smoked during pregnancy, and had fewer prenatal visits, ie, those at the highest risk of adverse outcomes.^19^ Another study reported that the likelihood of individuals with high risk in rural areas giving birth in a hospital with neonatal intensive care capacity depends on both geographic proximity and sociodemographic factors, including younger age, low income, and Black race.^20^

Our findings suggest that if Black individuals delivered at their nearest hospital, the disparity in care quality between Black and White individuals would be reduced. However, this does not imply that simply delivering at the nearest hospital is a comprehensive solution to reducing disparities. The US health care system is highly segregated, with significant disparities in hospital quality, referral systems for patients with high risk, and housing markets. Targeted interventions are needed to improve care quality at hospitals serving minoritized populations. Additionally, policies should focus on reducing segregation in health care and housing to ensure that high-quality care is accessible to all individuals, regardless of race or ethnicity.

### Limitations

This study has some limitations. Limitations include the lack of data on factors influencing the choice of delivery hospital and potential unmeasured confounders in our hospital quality risk adjustment. Additionally, we did not have access to geocoded addresses for individuals and calculated driving times based on the centroid of the residential zip code, which may introduce measurement error, particularly in larger zip code areas. However, this would only bias our findings if some racial or ethnic groups systematically lived farther from their zip code centroids than others. Since the distribution of residences near or far from the centroid is random, this limitation should not impact our results. Additionally, a study showed that driving distance from an individual’s geocoded address is highly correlated (*r* = 0.99) with the Euclidean distance from the zip code centroid.^37^

## Conclusions

In this cohort study, American Indian and Black birthing individuals delivered in lower-quality hospitals, whereas there was no significant difference for Asian and Hispanic individuals compared with White birthing individuals. Our findings also suggested that the disparity in care between Black and White birthing individuals would have been reduced if they had delivered at their nearest hospital. Addressing these disparities may require targeted interventions to improve access to high-quality hospitals for minoritized racial and ethnic groups and to address systemic issues that contribute to these disparities.

## Notes

Supplement 1. **eTable.** Adjustment Variables in the Obstetric Comorbidity Index **eFigure 1.** Study Cohort Exclusions **eFigure 2.** Racial Distribution Within Quintiles of Delivery Hospital Quality and Closest Obstetric Hospital Quality **eFigure 3.** Lorenz Curves for Inequality by Actual Delivery Hospital and Closest Obstetric Hospital Across All 5 States by Insurance Type

Supplement 2.
Data Sharing Statement
