# Supplementary material for: Racial and Ethnic Inequalities in Actual vs Nearest Delivery Hospitals
Source: JAMA Netw Open. 2025 Mar 21;8(3):e251404. doi: 10.1001/jamanetworkopen.2025.1404 (PMC11929030; doi:10.1001/jamanetworkopen.2025.1404)
Supplement: Supplement 1. — eTable. Adjustment Variables in the Obstetric Comorbidity Index eFigure 1. Study Cohort Exclusions eFigure 2. Racial Distribution Within Quintiles of Delivery Hospital Quality and Closest Obstetric Hospital Quality eFigure 3. Lorenz Curves for Inequality by Actual Delivery Hospital and Closest Obstetric Hospital Across All 5 States by Insurance Type [file jamanetwopen-e251404-s001.pdf]

Supplemental Online Content

Boghossian NS, Greenberg LT, Buzas JS, et al. Racial and ethnic inequalities in actual vs nearest delivery hospitals. *JAMA Netw Open*. 2025;8(3):e251404. doi:10.1001/jamanetworkopen.2025.1404

eTable. Adjustment Variables in the Obstetric Comorbidity Index

eFigure 1. Study Cohort Exclusions

eFigure 2. Racial Distribution Within Quintiles of Delivery Hospital Quality and Closest Obstetric Hospital Quality

eFigure 3. Lorenz Curves for Inequality by Actual Delivery Hospital and Closest Obstetric Hospital Across All 5 States by Insurance Type

This supplemental material has been provided by the authors to give readers additional information about their work.

© 2025 Boghossian NS et al. *JAMA Network Open*.

eTable 1. Adjustment variables in the obstetric comorbidity index

**Comorbidity**
Pulmonary hypertension
Chronic renal disease
Cardiac disease, preexisting
HIV/AIDS
Preeclampsia with severe features
Placental abruption
Bleeding disorder, preexisting
Anemia, preexisting
Twin/multiple pregnancy
Gestational age
Placenta previa, complete or partial
Neuromuscular disease
Asthma, acute or moderate/severe
Preeclampsia without severe features or gestational hypertension
Connective tissue or autoimmune disease
Uterine fibroids
Substance use disorder
Gastrointestinal disease
Chronic hypertension
Major mental health disorder
Preexisting diabetes mellitus
Thyrotoxicosis
Previous cesarean birth
Gestational diabetes mellitus
Delivery BMI $\geq$ 40
Maternal age

© 2025 Boghossian NS et al. *JAMA Network Open*.

eFigure 1. Distribution of crude hospital non-transfusion SMM rates per 10,000 deliveries for 3-year period (2008-2010) by state

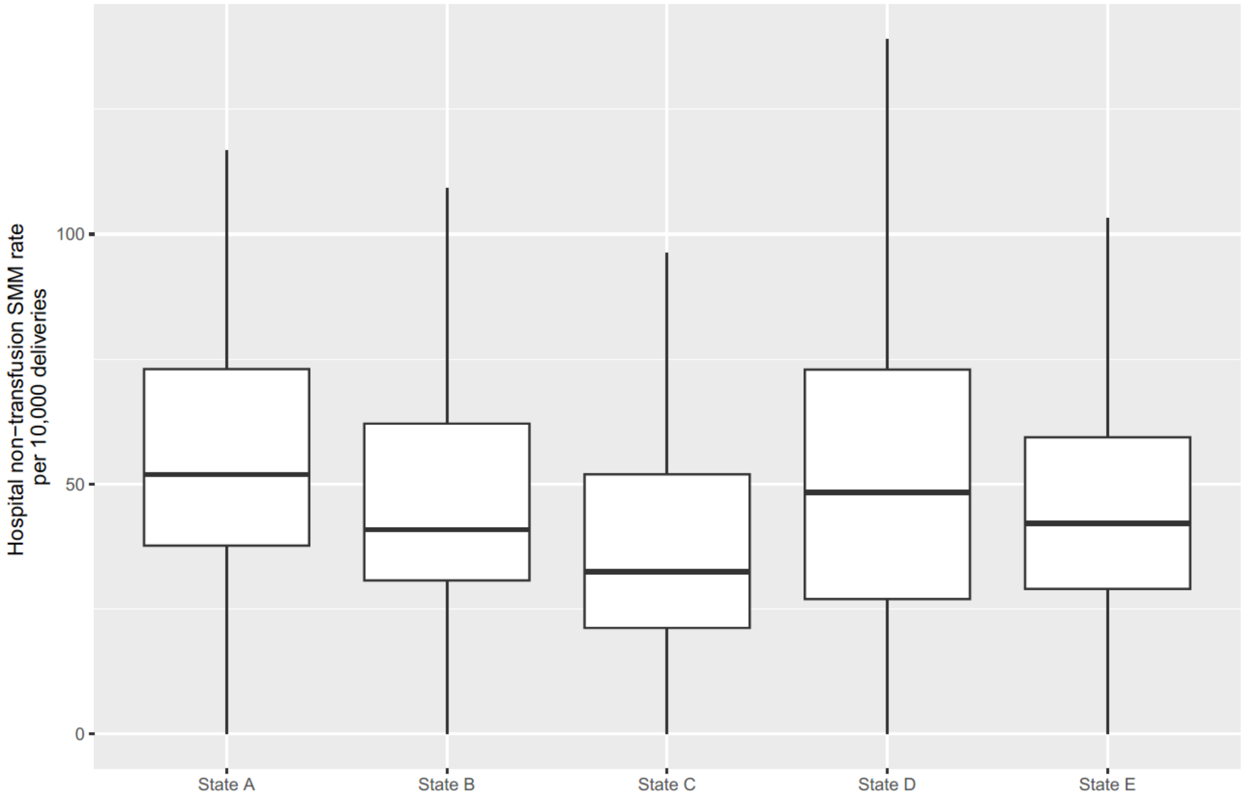

Lower border represents the 25th percentile of the data, upper border represents the 75th percentile of the data, line inside the box represents the 50th percentile of the data, whiskers represent the minimum and maximum values after excluding outliers (points > 1.5*IQR above or below the box).

	Percentile				
	10th	25th	50th	75th	90th
State A	32	38	52	73	101
State B	18	31	41	62	88
State C	0	21	32	52	65
State D	20	27	48	73	101
State E	21	29	42	59	81

© 2025 Boghossian NS et al. *JAMA Network Open*.

eFigure 2. Distribution of unadjusted and adjusted hospital-level standardized morbidity ratios (SMR) for non-transfusion SMM for a 3-year period (2008-2010) for all 5 states

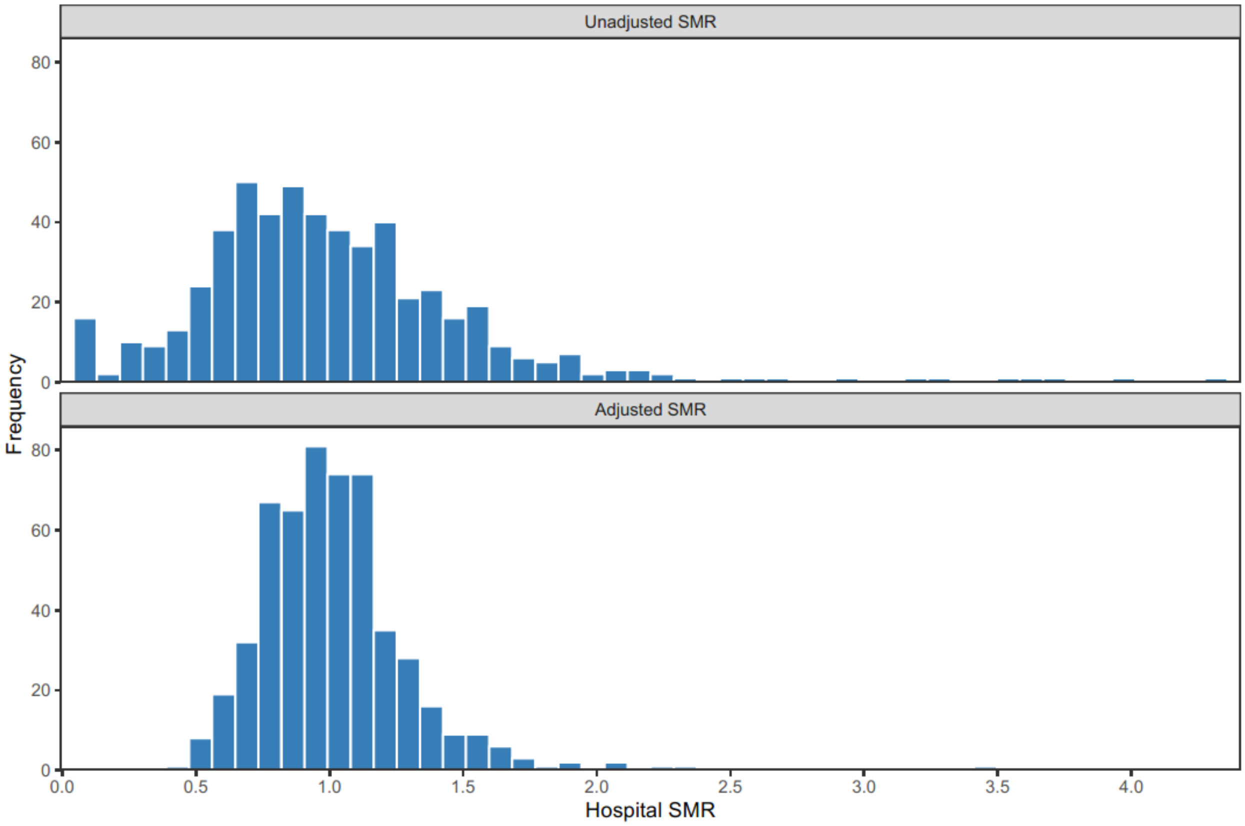

	Percentile				
	10th	25th	50th	75th	90th
Unadjusted SMR	0.5	0.7	0.9	1.2	1.6
Adjusted SMR	0.7	0.8	1.0	1.1	1.3

© 2025 Boghossian NS et al. *JAMA Network Open*.

eFigure 3. Study cohort exclusions

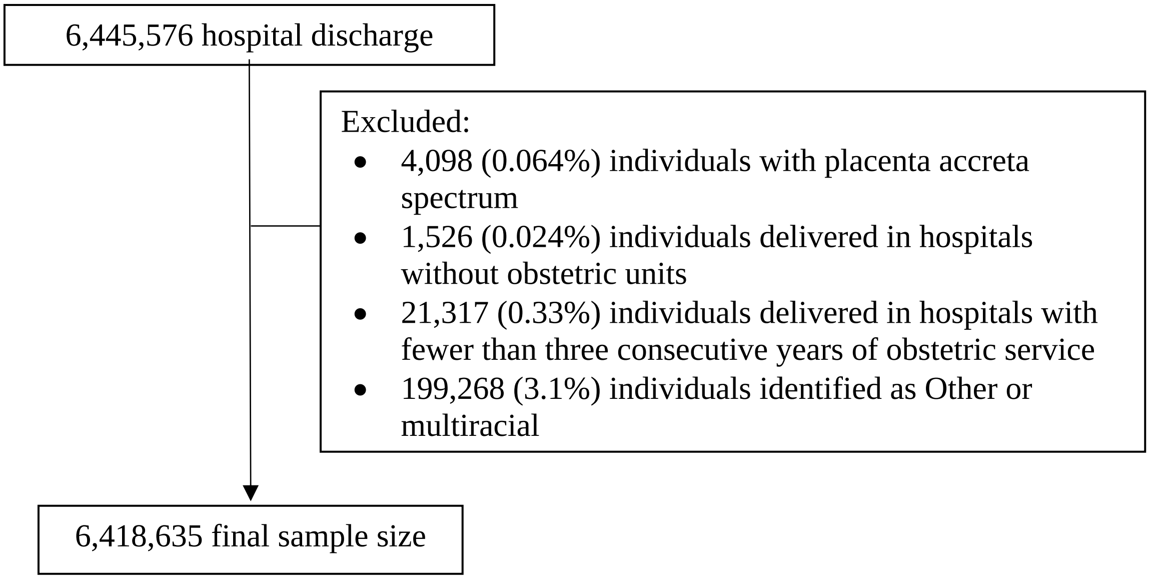

© 2025 Boghossian NS et al. *JAMA Network Open*.

eFigure 4. Racial distribution within quintiles of delivery hospital quality and closest obstetric hospital quality

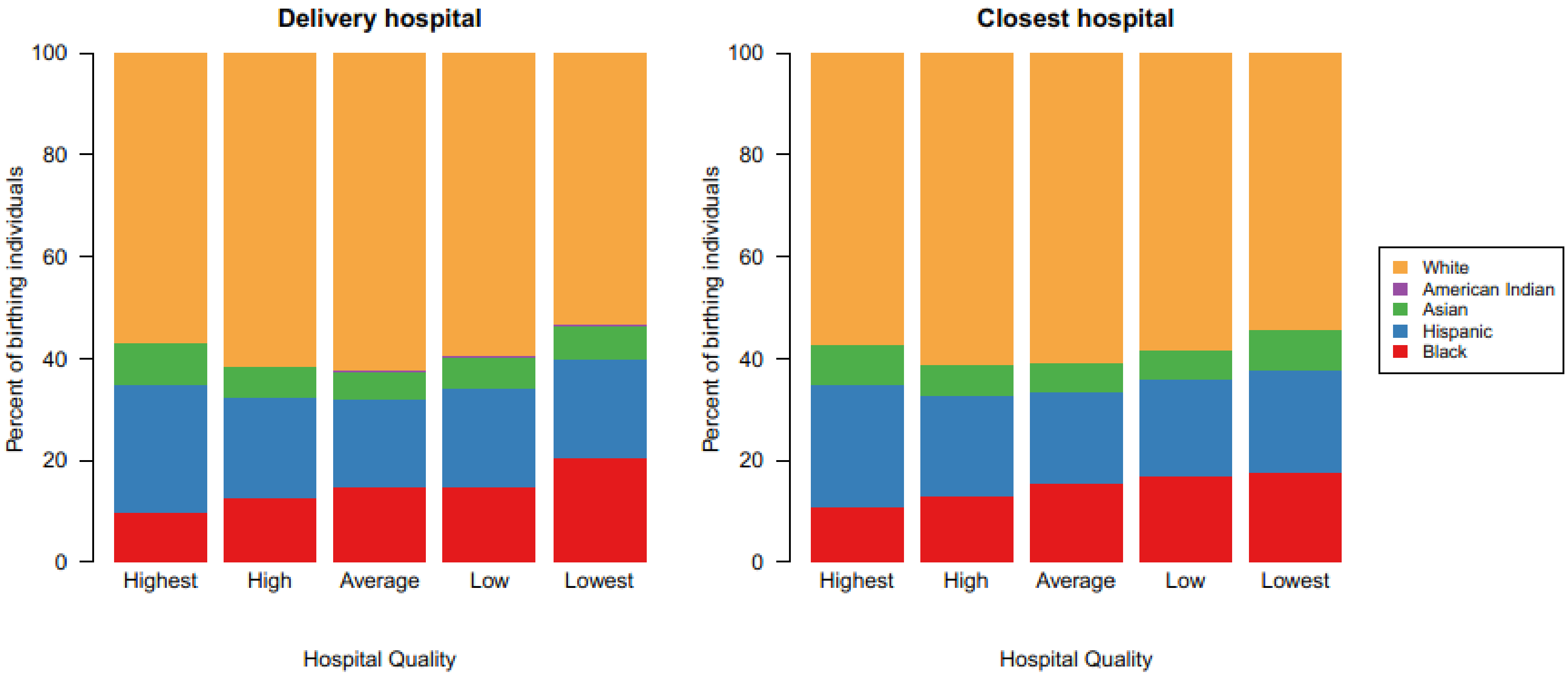

© 2025 Boghossian NS et al. *JAMA Network Open*.

eFigure 5. Lorenz curves for inequality by actual delivery hospital and closest obstetric hospital across all 5 states by insurance type; A: Government insurance, B: Commercial insurance

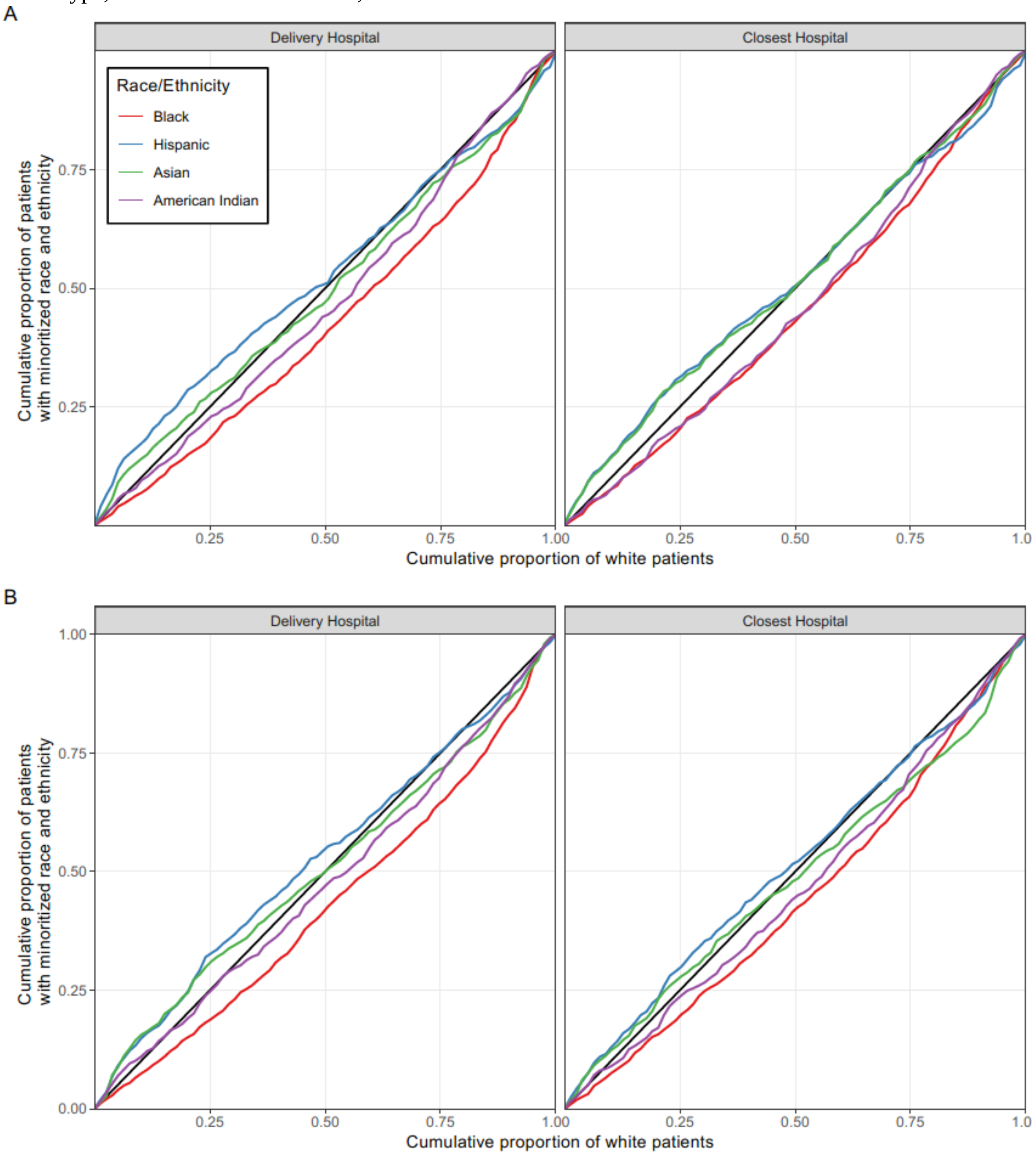

	Obstetric hospital inequality index (95% CI)			
	Black	Hispanic	Asian	American Indian
Government				
Delivery hospital	0.15 (0.10, 0.18)	-0.05 (-0.10, 0.02)	0.01 (-0.04, 0.08)	0.06 (0.01, 0.10)
Closest hospital	0.09 (0.06, 0.13)	-0.03 (-0.09, 0.03)	-0.03 (-0.08, 0.02)	0.07 (0.02, 0.12)
Commercial				
Delivery hospital	0.14 (0.10, 0.18)	-0.05 (-0.10, 0.00)	-0.01 (-0.09, 0.06)	0.05 (0.00, 0.10)
Closest hospital	0.12 (0.07, 0.15)	-0.03 (-0.07, 0.02)	0.03 (-0.02, 0.09)	0.07 (0.02, 0.12)

© 2025 Boghossian NS et al. *JAMA Network Open*.
