# Supplementary material for: Racial and Ethnic Inequalities in Actual vs Nearest Delivery Hospitals
Source: JAMA Netw Open. 2025 Mar 21;8(3):e251404. doi: 10.1001/jamanetworkopen.2025.1404 (PMC11929030; doi:10.1001/jamanetworkopen.2025.1404)
Supplement: Supplement 2. — Data Sharing Statement [file jamanetwopen-e251404-s002.pdf]

Data Sharing Statement

Boghossian. Racial and Ethnic Inequalities in Actual vs Nearest Delivery Hospitals. *JAMA Netw Open*. Published March 21, 2025. doi:10.1001/jamanetworkopen.2025.1404

Data
Data available: No

Additional Information
Explanation for why data not available: The data have been acquired from the states through data use agreements.
